# The effects of bioactive components from the rhizome of *gastrodia elata blume* (Tianma) on the characteristics of Parkinson’s disease

**DOI:** 10.3389/fphar.2022.963327

**Published:** 2022-11-30

**Authors:** Changcheng Lu, Shuhui Qu, Zhangfeng Zhong, Hua Luo, Si San Lei, Hai-Jing Zhong, Huanxing Su, Yitao Wang, Cheong-Meng Chong

**Affiliations:** State Key Laboratory of Quality Research in Chinese Medicine, Institute of Chinese Medical Sciences, University of Macau, Macao, China; International Cooperative Laboratory of Traditional Chinese Medicine Modernization and Innovative Drug Development of Chinese Ministry of Education (MOE), College of Pharmacy, Jinan University, Guangzhou, China; Macau Centre for Research and Development in Chinese Medicine, State Key Laboratory of Quality Research in Chinese Medicine, University of Macau, Macao, China

**Keywords:** Parkisnon’s disease, tianma (Gastrodia elata blume), dopaminergic neurons, neuroinflmamation, synuclein

## Abstract

Parkinson’s disease (PD) is an age-related chronic neurodegenerative disease caused by the death and degeneration of dopaminergic neurons in the substantia nigra of the midbrain. The decrease of the neurotransmitter dopamine in the patient’s brain leads to various motor symptoms. PD drugs mainly enhance dopamine levels but cannot prevent or slow down the loss of dopaminergic neurons. In addition, they exhibit significant side effects and addiction issues during long-term use. Therefore, it is particularly urgent to develop novel drugs that have fewer side effects, can improve PD symptoms, and prevent the death of dopaminergic neurons. The rhizome of *Gastrodia elata Blume* (Tianma) is a well-known medicinal herb and has long been used as a treatment of nervous system-related diseases in China. Several clinical studies showed that formula comprising Tianma could be used as an add-on therapy for PD patients. Pharmacological studies indicated that Tianma and its bioactive components can reduce the death of dopaminergic neurons, α-synuclein accumulation, and neuroinflammation in various PD models. In this review, we briefly summarize studies regarding the effects of Tianma and its bioactive components’ effects on major PD features and explore the potential use of Tianma components for the treatment of PD.

## Introduction

Parkinson’s disease (PD) is a common chronic neurodegenerative disease, which results in motor symptoms such as bradykinesia, stiffness, resting tremor, and postural instability (Kalia and Lang, 2015). Most PD symptoms are caused by the degeneration and loss of dopaminergic neurons that produce dopamine in the substantia nigra (SN) of the midbrain (Dickson et al., 2009). Reduced dopamine level results in abnormal brain transmission activity, leading to impaired movement and various motor symptoms. PD also leads to non-motor symptoms such as dizziness, cognitive changes, psychiatric and sleep problems. PD affects individuals all over the globe and is the second most prevalent neurological illness after Alzheimer’s disease. According to the statistics of PD patients worldwide from 1990 to 2015, the prevalence, disability-adjusted life-years, and deaths increased substantially, making PD the fastest-growing neurological disease (Feigin et al., 2017).

The incidence of PD increases with age, particularly in elderly individuals over 50 years of age (Bloem et al., 2021). With the increase of the aging population, the prevalence of PD will continue to grow and is expected to exceed 12 million by 2040 (Dorsey and Bloem, 2018). Meta-analysis suggests that there are significant differences in PD prevalence by geographical location. The prevalence rates in North America, Europe, and Australia are 2.4 times higher than that of Asia. In addition, the prevalence in women is three-tenths of that in men (Pringsheim et al., 2014). Environmental risk factors for PD are also identified through epidemiology. Environmental chemicals such as the pesticide paraquat are found to be a risk factor in the development of PD (Clarimon, 2020). PD is a neurological ailment that worsens with age; thus, aging is considered the major driver of PD development (Antony et al., 2013). However, since PD’s etiology and pathogenic mechanisms are still poorly understood, it is challenging to develop drugs to prevent the death of dopaminergic neurons. Currently, PD drugs including levodopa preparations, dopamine receptor agonists, and monoamine oxidase inhibitors mainly work through enhancing dopamine levels, but they exhibit a large number of side effects and addiction issues (Bastide et al., 2015). Conversely, they may generate excessive dopamine action, causing the abnormal activation of the motor system and thereby generating dyskinesias. The standard oral levodopamine (l-dopa) treatment is the most effective way to relieve PD patients’ motor symptoms. However, as the disease progresses and l-dopa continues to be taken, the movement disorder caused by the drug l-dopa, known as l-dopa-induced dyskinesia (LID), are often seen in patients who are on the treatment for over 10 years (Bastide et al., 2015). Notably, these PD drugs cannot prevent or slow down the death of dopaminergic neurons. Therefore, it is particularly urgent to find drugs that have fewer side effects, can improve the PD symptoms, and prevent the death of dopaminergic neurons.

**FIGURE 1:**
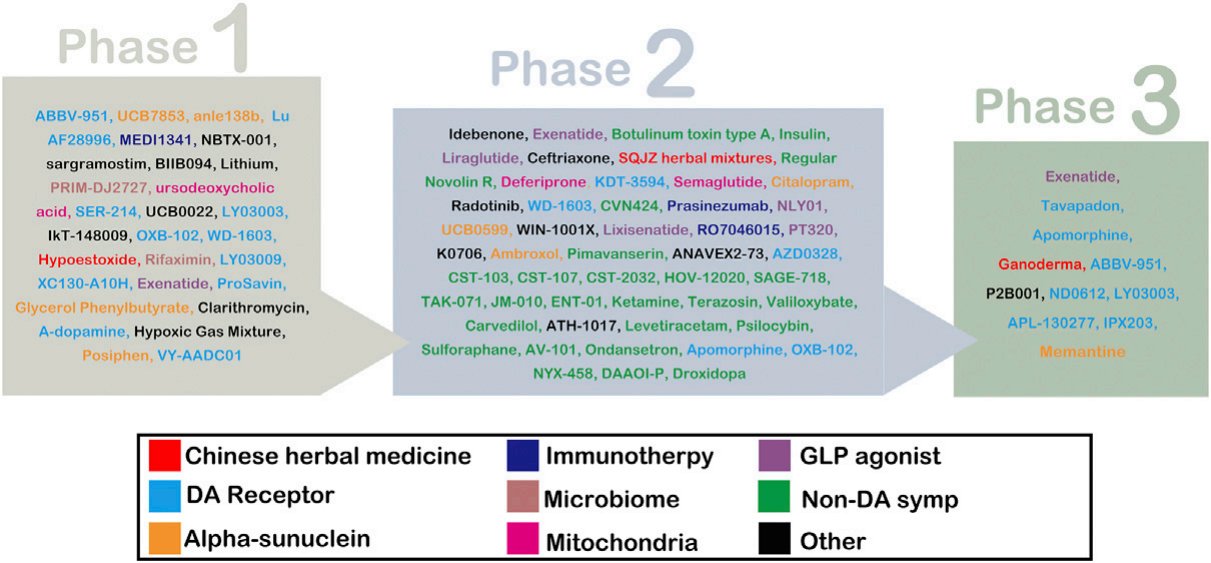
List of drug-modifying therapies and symptomatic therapies for PD.

Up to now, many medicinal herbs or natural products have been developed as PD drug candidates. Some have entered in clinical trials (Figure 1) (Prasad and Hung, 2021). For example, Ganoderma (NCT03594656) has entered phase III of a trial. And the SQJZ herbal mixture (NCT02616120), a formula consisting of Cornus officinalis, Rehmannia glutinosa, Poria cocos, Ophiopogon japonicus, Cuscuta chinensis, Trichosanthes kirilowii, Semen ziziphin spinosae, Aurantii fructus immaturus, and Schisandra chinensis (Shi et al., 2017), has entered phase II. Additionally, Hypoestoxide (NCT04858074), a natural diterpenoid found in Hypoestes rosea dry leaf powder, is in phase I. These imply that medicinal herbs may have potential in the treatment of PD without serious side effects.

**FIGURE 2:**
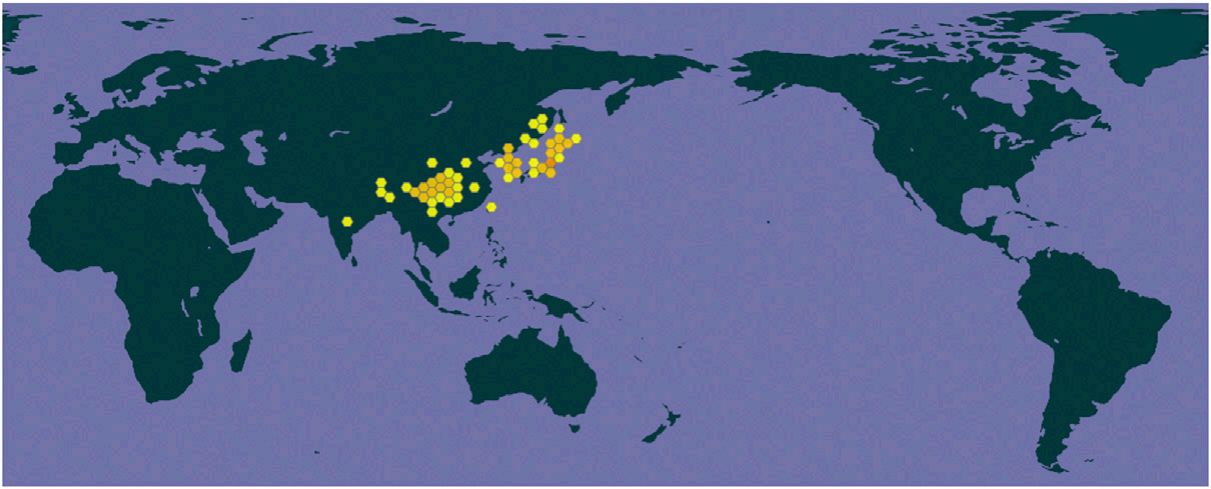
The origin of *Gastrodia elata blume* (Tianma).

*Gastrodia elata blume* (Tianma) is a medicinal herb first found in the Classic of the Materia Medica, also known as the *Shennong Ben Cao Jing* (Zhan et al., 2016; Zhu et al., 2019; Heese, 2020). According to the Global Biodiversity Information Facility database (https://www.gbif.org/), Tianma has 20 synonyms and belongs to the genus *Gastrodia R. Br.* Family Orchidaceae. It is mainly distributed in the mountainous regions of eastern Asia, especially in Korea, Japan, China, and India (Figure 2) (Gbif Global Biodiversity Information Facility, 2022). In ancient China, this was the preferred remedy to benefit and strengthen the qi, nourish the yin, and strengthen the body and prolong life (Zhan et al., 2016). Nowadays, it is used clinically for the treatment of dizziness, spasms, epilepsy, headache, amnesia, and hypertension (Matias et al., 2016; Zhan et al., 2016). Several clinical studies showed that the famous formula Tianma Gouteng decoction could be used as an add-on therapy for PD patients (Chen et al., 2014; Yan et al., 2014; Liu, 2015; Zhao and Hu, 2017; Zuo, 2018; Wang et al., 2019; Li et al., 2020). Furthermore, a growing number of studies have shown that Tianma and its constituents show neuroprotective effects in various PD *in vitro* and *in vivo* models. The main bioactive components of Tianma such as gastrodin, vanillyl alcohol, vanillin, vanillic acid, and anisalcohol have neuroprotective activities, which have attracted strong interest in the treatment of PD. Although the neuroprotective effects of the extracts and some compounds of Tianma have been summarized in previous reviews, new evidence from recent studies support the multiple activities of Tianma in specific PD features. Thus, in this review, we update and briefly summarize studies regarding the effects of the bioactive components of Tianma on the main features of PD and explore their potential for the treatment of PD.

## The characteristics of PD brain

### Dopaminergic neuronal death

**FIGURE 3:**
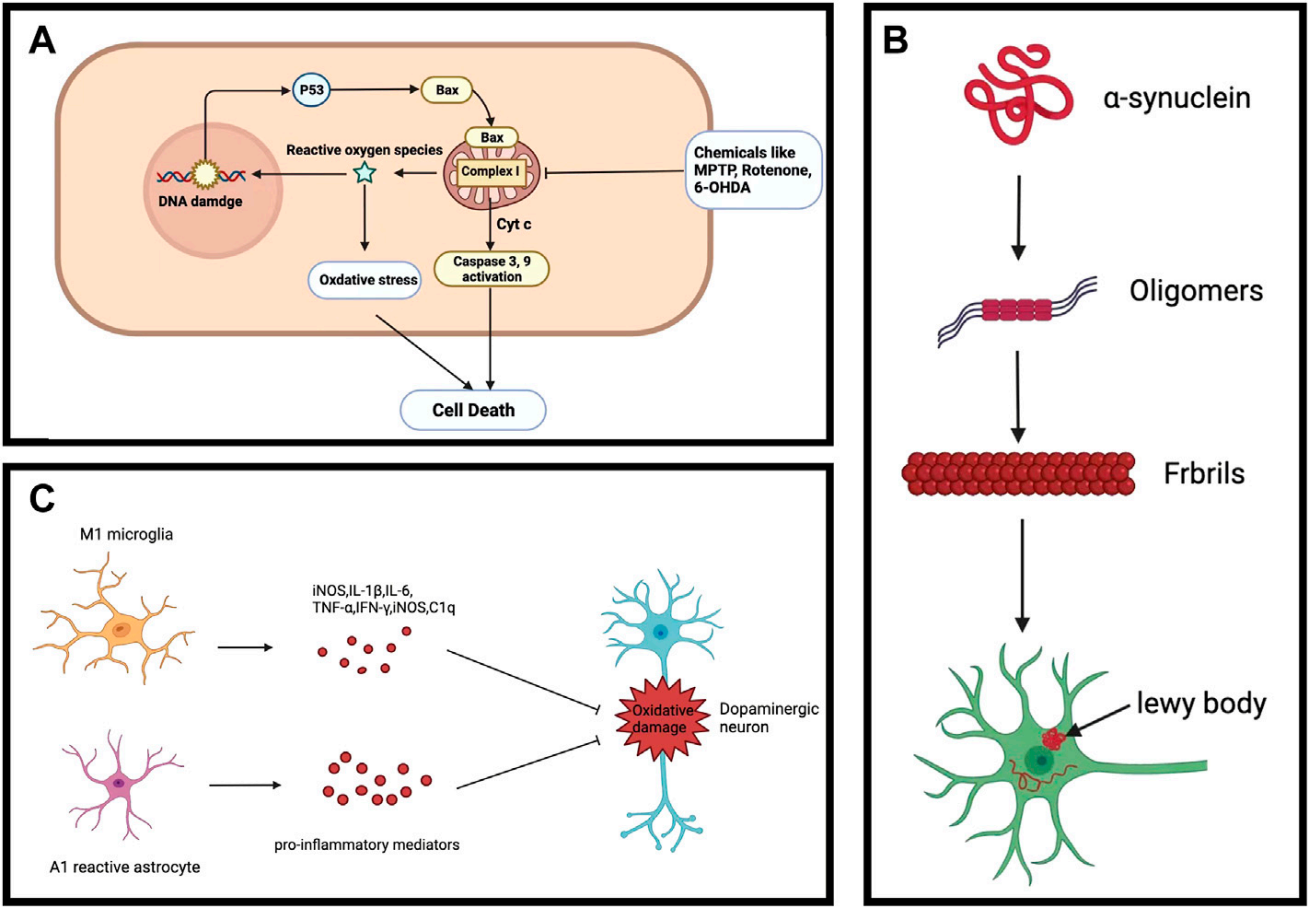
Common PD characteristics. **(A)** Dopaminergic neuronal death. **(B)** Alpha-synuclein aggregation. **(C)** Neuroinflammation.

The main pathological feature of PD is the decrease in dopamine caused by the death of dopaminergic neurons (Figure 3A). Although it is unknown what causes the dopaminergic neurons to die, dopaminergic neurodegeneration is accompanied by mitochondrial dysfunction and increase in reactive oxygen species (ROS) (Poewe et al., 2017). Mitochondrial dysfunction has been considered as one of the leading causes of dopaminergic neuron death. The mitochondria is an organelle found in the cytoplasmic matrix that generates energy *via* the mitochondrial respiratory chain (Bose and Beal, 2016), which serve an important role in metabolism, storing the intermediate products of pyruvate oxidation and Krebs cycle. Mitochondria also participates in calcium homeostasis, free radical scavenging, and programmed cell death control (Feno et al., 2019). However, other than powering and maintaining cellular function, the mitochondria is also the main source of ROS generation.

The functional changes of mitochondria are manifested in PD patients, pointing toward a critical, causal role of mitochondrial defects in the degeneration of dopaminergic neurons (Cui et al., 2012). Notably, deficiency in mitochondrial complex I was observed in the SN of PD patients (Perier et al., 2010). The *Ndufs4* gene encodes the core subunit protein of reduced nicotinamide adenine dinucleotide (NADH) dehydrogenase (complex I) in the mitochondrial membrane respiratory chain. A recent study indicated that knocking out the *Ndufs4* gene in mice could cause mitochondrial complex I dysfunction and was sufficient to induce human-like PD symptoms (Gonzalez-Rodriguez et al., 2021). In addition, a vicious cycle between increased cytosolic dopamine and elevated mitochondrial oxidative stress exists in PD neurons (Gegg and Schapira, 2016; Burbulla et al., 2017; Nguyen et al., 2019). A myriad of studies has shown that toxin 1-methyl-4-phenyl-1,2,3, 6-tetrahydropropyne (MPTP), rotenone, 6-hydroxydopamine (6-OHDA), pyridine toluene, trichloroethylene, and fenpyrrolazine, which are complex I inhibitors, can lead to neurodegeneration of dopaminergic neurons in animal models. These inhibitors result in increased ROS production from mitochondrial dysfunction, which contributes to the activation of the intracellular apoptotic pathway: Bax is translocated to the mitochondria and cytochrome c is released from the mitochondria into the cell matrix, triggering a caspase activation cascade, leading to cell death. On the other hand, ROS induces the activation of poly ADP-ribose polymerase (PARP), which consumes adenosine triphosphate (ATP) and nicotinamide adenine dinucleotide (NAD+), leading to programmed cell death (Bose and Beal, 2016). Enriched ROS and Ca^2+^ in mitochondria can cause mitochondrial swelling and dysfunction. Although it is controversial whether oxidative stress caused by mitochondrial dysfunction occurs at an early or late stage of PD (Dionisio et al., 2021), accumulated evidence supports that mitochondrial complex I defects are nevertheless involved in the loss of dopaminergic neurons.

### Lewy bodies

Lewy bodies (LBs) are mainly composed of aggregated presynaptic neuron protein α-synuclein (α-Syn), whose presence is a characteristic pathologic feature of PD. In addition to the SN, LBs also occur in other areas of the midbrain, basal forebrain, and cerebral cortex as PD develops. Misfolding and aggregation of α-syn involve seeding and nucleation mechanisms and are major causes of LBs formation in PD neurons (Figure 3B). However, it is unknown what is the exact conditions that lead to aberrant misfolding and aggregation of α-Syn in PD. α-Syn aggregation was reported to be linked to proteostasis dysfunction, such as chaperone-assisted proteolysis, autophagy, and ubiquitin-proteasome system -mediated degradation (Mehra et al., 2019). Notably, loss-of-function mutations in lysosomal beta-glucocerebrosidase (GCase) result in the accumulation of α-Syn (Migdalska-Richards and Schapira, 2016). On the other hand, non-inhibitory small-molecule modulators of GCase specifically enhanced lysosomal compartment activity, leading to a reduction in GCase substrate and clearance of pathological α-Syn in human PD patient midbrain neurons (Mazzulli et al., 2016). These suggest that lysosomal degradation plays a key in the accumulation of α-Syn. Additionally, mice overexpressing α-Syn are more sensitive to MPTP toxicity (Lee et al., 2017), suggesting that α-Syn accumulation may increase the vulnerability to PD. Although the LBs do not directly lead to dopaminergic neuronal death, the accumulation of α-Syn can act as an indicator for evaluating the effects of potential drug candidates.

### Neuroinflammation

Another important aspect of PD is chronic neuroinflammation. Neuroinflammation in PD is mostly linked to the reactive state of glial cells (astrocytes and microglia) in the brain. Since glial cells have bidirectional communication with neurons, loss of dopaminergic neurons is accompanied by a significant increase in the number of reactive glial cells in the SN in PD patients (Mani et al., 2021). Microglia are the primary glial cells for mediating the innate immune response against invading pathogens and scavenging damaged neurons, synapses, as well as abnormally aggregated proteins in the central nervous system through phagocytosis (Harry, 2021; Zhong et al., 2022). However, alterative immune response of microglia can be activated by secreted molecules from degenerative neurons, which leads to the secretion of pro-inflammatory cytokines such as Interleukin-1β (IL-1β), IL-6, and tumor necrosis factor-α (TNF-α) to the brain microenvironment (Figure 3C). These pro-inflammatory cytokines may accelerate neurodegeneration in PD. Reducing microglia-mediated neuroinflammation can thus decrease the degeneration of dopaminergic neurons and improve motor behavior problems (Meng et al., 2019). Although the real cause of neuroinflammation is unclear, aggregated α-synuclein, mitochondrial dysfunction, and ecological dysregulation of the gut microbiome may induce neuroinflammation (Wang et al., 2021). Given the vital role of neuroinflammation in the progression of PD, regulating it may improve the brain microenvironment and reduce neurodegeneration.

## The common experimental models of PD

MPTP is a Parkinsonism-inducing neurotoxin. It is a highly lipophilic molecule that can cross the blood-brain barrier (BBB) and specifically destroy dopaminergic neurons, thus MPTP is commonly used for establishment of various animal PD models (Ke et al., 2021). *In vitro* PD models based on dopaminergic neuronal death are commonly used for identifying PD drug candidates. 1-Methyl-4-phenylpyridinium ion (MPP^+^) is a metabolite of MPTP catalyzed by monoamine oxidase-B (MAO-B) in the brain. It can be specifically taken up by dopaminergic neurons and neuron-like cells, which causes injury to the mitochondria and thereby leads to apoptosis. SHSY-5Y and the MN9D cell lines are commonly used in the MPP^+^ cell model since they have the ability to synthesize and release dopamine (Xie et al., 2010). They also express tyrosine hydroxylase and dopamine transporter protein (DAT) (Xie et al., 2010). In addition, these cell lines are treated with 6-OHDA and H_2_O_2_ to induce oxidative stress, which is a causative factor for dopaminergic neuronal death in PD (Gay et al., 2018). The changes in apoptosis-related markers such as Bcl-2, Bax, and caspases three are major indicators for evaluating the effects of drug candidates on cell death (Abushouk et al., 2017).

LPS-induced microglial activation is a classical model for identifying drug candidates to evaluate anti-neuroinflammation properties (Catorce and Gevorkian, 2016). BV-2 is a microglial cell line derived from C57/BL6 mice. After LPS treatment, it enters the M1 polarised state, which is the main cause of neuroinflammation in PD. The changes in proinflammatory factors such as nitric oxide (NO), TNF-α,IL-1β, and COX-2 are common indicators for evaluating the effects of drug candidates on neuroinflammation.

## The therapeutic effects of chinese medicine formula containing tianma

According to the ancient record of Chinese medicine, Tianma Gouteng decoction is a well-known formula containing Tianma, Uncaria Ramulus Cum Uncis, Haliotidis Concha, Gardeniae Fructus, Scutellariae Radix, Cyathulae Radix, Leonur Iherba, Eucommiae Cortex, Taxilli Herba, Caulis Polygoni Multiflori, and Zhu Fushen, originally used in the treatment of various disorders such as hypertension, migraine (Chang et al., 2014; Chong et al., 2021), and dementia (Lin et al., 2015). In preclinical studies, this formula inhibited lipid peroxidation by attenuating the lipid peroxidation-related protein ALOX15, thereby improving behavioral deficits and reducing the loss of dopaminergic neurons in MPTP-induced mice, 6-OHDA-induced rat, and α-Syn A53T overexpressed mice (Jiang Y.N et al., 2020). In clinical studies, Tianma Gouteng decoction was found to be a potential candidate for the treatment of PD. Its therapeutic effects are based on usage in combination with clinical drugs such as selegiline, levodopa, medopar, or pramipexole (Chen et al., 2014; Yan et al., 2014; Liu, 2015; Zhao and Hu, 2017; Zuo, 2018; Wang et al., 2019; Li et al., 2020). Compared to single treatment with these drugs, the combined treatment with Tianma Gouteng decoction leads to significantly lower scores in Parts I, II, III, and IV of the Unified PD Rating Scale (UPDRS). The treatment of Tianma Gouteng Decoction combined with selegiline decreased the serum levels of TNF-α, IL-6, malondialdehyde (MDA), and Cys-C when compared to the control group (Zuo, 2018). In the treatment of Tianma Gouteng decoction combined with Levodopa, it was found that the levels of IL-2, IL-6, TNF-α, MDA, and Cys-C in the treatment group decreased significantly and the SOD level increased significantly after 3 months (Gu et al., 2018). In the treatment of Tianma Gouteng decoction combined with Madopar, the levels of Glu and GABA in the treatment group were significantly increased compared to the control group (Liu et al., 2020). Serum homocysteine (Hcy) decreased, serum uric acid (UA) and adiponectin (APN) levels increased in Tianma Gouteng decoction combined with pramipexole treatment (Liu, 2020; Gui, 2021). These clinical evidences support that Tianma Gouteng decoction can be used as an add-on therapy for improving PD motor symptoms and blood biochemical parameters in PD.

## The effects of tianma extract on various PD models

Tianma is a major medicinal herb in Tianma Gouteng decoction, hinting that it may have some potential therapeutic effects for PD. Evidence suggests that Tianma display strong antioxidant properties and neuroprotection in *vitro* and *in vivo* PD models. In MPP^+^-treated SH-SY5Y cells, the ethanol extract of Tianma conferred protective effects against MPP^+^-induced cell death (An et al., 2010). Researchers also demonstrated that the water extract of Tianma is an effective treatment for alleviating the motor symptoms of parkinsonian and loss of dopaminergic neurons in the fly model with LRRK2-G2019S mutation, which is the most common familial PD mutation (Lin et al., 2021). They found that the extract of Tianma activated the Akt-Nrf2 pathway in glia but not in neurons, contributing to its neuroprotective effects. Simultaneously, the extract of Tianma also worked as a neuroprotectant to improve locomotor performance, reduce dopaminergic neuron loss, and microglial activation in mice with LRRK2-G2019S mutation (Lin et al., 2021). Tianma extract could reduce lipopolysaccharides (LPS)-stimulated neuroinflammation BV-2 microglia model by down-regulating JNK/nuclear factor-κB (NF-κB) pathway (Kim et al., 2012), supporting the anti-neuroinflammation activity of Tianma. In 6-OHDA mice, l-dopa’s adverse effect LID was alleviated by Tianma extract *via* normalizing FosB and ERK activation (Doo et al., 2014). On the other hand, GABA levels in the SN pars reticulata can be increased by high-frequency stimulation of the subthalamic nucleus which is a therapy for patients with severe PD (Windels et al., 2005). Tianma extract could improve cognitive impairment and recover normal GABA levels in aluminum-treated rats (Shuchang et al., 2008). Tianma also provides anxiolytic-like effects *via* regulating the GABAergic nervous system (Ha et al., 2000). Taken together, due to the multiple activities of Tianma, we believe the bioactive components from Tianma may have the potential to alleviate various PD characteristics and symptoms.

## The effects of bioactive compounds of tianma on various PD models

To present, at least 81 compounds have been isolated from Tianma, with five major groups including phenols and their glycosides, polysaccharides, sterols, organic acids, and other compounds (Zhu et al., 2019). The contents of these compounds are comprised of total phenolics (0.0485%), total polysaccharides (21.6%), total flavonoids (0.235%), total amino acids (1.92%), β sitosterol (0.113%), and other undetermined compounds (77.8%) (Zhan et al., 2016). Amongst them, the bioactive components of Tianma include gastrodin, vanillyl alcohol, vanillin, vanillic acid, and anisalcohol, which exhibit neuroprotective activities in various PD models. Gastrodin is considered as the major reference standard and the main bioactive component of Tianma. The maximum content of gastrodin reaches 0.24%, while other active components such as vanillin, vanillyl alcohol, vanillic acid, and anisalcohol belong to phenolic compounds that in total reach 0.0485% detected by the Folin-Ciocaileu colorimetric method (Zhan et al., 2016). The EtOEt extract of Tianma was subjected to the phenolic separation of compounds and showed to comprise Vanillyl Alcohol (0.00195%), Vanillin (0.00282%), and Vanillic acid (0.00195%) (Jang et al., 2010). Another report found that Tianma extracted with ethyl acetate to obtain the ethyl acetate fraction of Tianma after the crude extract of ethanol gives rise to anisyl alcohol (0.009445%) (Duan et al., 2013).

### Gastrodin

Gastrodin (4-hydroxybenzyl alcohol 4-O-bata-d-glucoside) is a major active compound found in Tianma. The potential of gastrodin in various PD models attracts scientists’ strong attention. Pretreatment of gastrodin inhibited apoptosis in MPP^+^/SH-SY5Y cells model through modulating oxidative stress, cleaved PARP, Bax/Bcl ratio, and caspase-3 (Kumar et al., 2013). Some reports showed that gastrodin reduced MPP^+^-induced death of human dopaminergic SH-SY5Y cells through a reduction in Bcl-2/Bax ratio and ROS production (Jiang et al., 2014). Gastrodin increased the Nrf2 nuclear translocation and further enhanced heme oxygenase-1 (HO-1) expression *via* the p38 mitogen-activated protein kinase (MAPK) pathway. Pretreatment of rat glioma cell line C6 with gastrodin could reduce hydrogen peroxide-induced ferroptosis, which is a type of necrosis caused by iron-induced accumulation of lipid hydroperoxide (Jiang T et al., 2020). In this model, gastrodin decreased released lactate dehydrogenase (LDH) and intracellular MDA level. It up-regulated the expression of nuclear Nrf2, glutathione peroxidase 4 (GPX4), ferroportin-1 (FPN1), and HO-1, contributing to its cytoprotective effects against hydrogen peroxide-induced oxidative injury. Gastrodin can penetrate the BBB (Lin et al., 2007); thus it is well studied in various PD animal models. Others reported that gastrodin could ameliorate MPTP-induced motor deficits of mice in the open-field test and the rotating test (Wang et al., 2014). Oxidative stress in MPTP-induced PD mice was reduced by the antioxidant effects of gastrodin. In the striatum of MPTP mice, gastrodin not only robustly enhanced the expression of HO-1 and superoxide dismutase (SOD), but also promoted Nrf2 nuclear translocation (Wang et al., 2014). They further found that ERK1/2 phosphorylation contributes to the Nrf2 antioxidant system activated by gastrodin. Nrf2-mediated the expression of antioxidants and detoxification proteins, which are major antioxidant responses to defend against ROS, linking neuroprotective effects of gastrodin to the ability to enhance intracellular antioxidant responses. In 6-OHDA rat model of PD, pretreatment with gastrodin not only significantly improved motor incoordination and rigidity but also reduced lipid peroxidation levels, NO production, and increased total antioxidant capacity in the SN. In a rotenone-induced PD rat model, gastrodin could improve motor deficits and reduced the death of dopaminergic neurons in the SN (Li et al., 2012). Others found that gastrodin enhanced the food clearance as well as decreased pumping rate and the loss of dopaminergic neurons in the 6-OHDA *Caenorhabditis elegans* (C. elegans) model of PD *via* the DAF-2/DAF-16 insulin-like pathway (Yan et al., 2019). Pink1^B9^ mutant Drosophila can generate various PD-like phenotypes. Gastrodin significantly prolonged lifespan and enhanced the climbing ability in this PD fly model (He et al., 2021). In addition, gastrodin treatment increased the dopamine level and rescued the progressive death of dopaminergic neurons in the protocerebral posterial lateral one region of the PD fly brain (He et al., 2021). Notably, gastrodin also extended the lifespan, physical activity, and antioxidant ability in wild-type Drosophila, revealing that gastrodin may display anti-aging activity.

Reactive microglia-mediated neuroinflammation plays a role in the pathological process of PD (Li et al., 2012). In LPS-induced bv-2 microglia, gastrodin can be found to reduce the expression of pro-inflammatory cytokines such as COX-2 and NO by inhibiting MAPK phosphorylation and the NF-κB signaling pathway (Dai et al., 2011). Another report also showed that gastrodin decreased the expression and production of pro-inflammatory cytokines in LPS-induced bv-2 microglia by inhibiting the Wnt/β-catenin signaling pathway (Yao et al., 2019). Neuroinflammation parameters such as increased pro-inflammatory cytokines IL-1β, IL-6, and TNF-α as well as decreased resting microglia are observed in the SN of rotenone-induced PD rat, whereas gastrodin could decrease them. Myeloperoxidase (MPO) is a critical peroxidase enzyme increased in reactive microglial and contributes to the degeneration of dopaminergic neurons (Chen et al., 2020). In 6-OHDA-induced PD rat model, MPO activity in the SN was reduced by gastrodin (Haddadi et al., 2018), revealing that gastrodin has the effects in reducing microglial activation in various PD models. Additionally, gastrodin was found to attenuate α-Syn accumulation in α-Syn overexpressing transgenic worms (Yan et al., 2019). These pieces of evidence support that gastrodin can enhance the intracellular antioxidant system, reduce neuroinflammation and α-Syn accumulation, and contribute to reducing the death of dopaminergic neurons and PD-like motor problems.

### Vanillin

Vanillin (4-hydroxy-3-methoxybenzaldehyde), a fragrant organic phenolic molecule, is widely used as a flavoring agent in food, beverage, cosmetics, and pharmaceutical industries. Vanillin is also identified in Tianma and can cross BBB (Salau et al., 2020). It has been reported to provide neuroprotection in different *in vivo* PD neurotoxin models. In a rotenone-induced PD rat model, improvements in both motor and depressive like behavior were observed after vanillin treatment. Vanillin could reduce rotenone-induced striatal dopamine depletion and oxidative stress. It also reduced rotenone-induced changes of apoptotic protein markers such as increased cytochrome C, decreased Bax, and increased Bcl-2 (Dhanalakshmi et al., 2016). In 6-OHDA PD rats, vanillin treatment could significantly decrease apomorphine-induced tight contralateral rotation and maintain dopamine levels (Abuthawabeh et al., 2020). In addition, pretreatment of vanillin could attenuate rotenone-induced mitochondrial dysfunction, oxidative stress, and apoptotic cascade in human SH-SY5Y cells (Dhanalakshmi et al., 2015).

Moreover, anti-neuroinflammatory effect of vanillin is mainly mediated *via* microglial activation, which has been demonstrated both *in vivo* and *in vitro*. LPS leads to motor dysfunction and dopaminergic neuron death in the SN of mice, whereas vanillin decreases neuroinflammation. Vanilin reduces LPS-induced expression of cyclooxygenase-2 (COX-2), inducible NO synthase (iNOS), and pro-inflammatory factors such as IL-1β and IL-6 through modulation of p38, ERK1/2, and NF-κB signaling (Yan et al., 2017). An *in vitro* study showed that vanillin inhibits the phosphorylation of MAPKs and NF-κB in LPS-treated microglia, and also reduces the protein levels of COX-2 and iNOS, as well as the mRNA levels of IL-1β, TNF-α and IL-6 (Kim et al., 2019). These reports suggest that vanillin is a potential drug candidate for the prevention of PD-related neuroinflammation through the inhibition of microglial activation. Thus, the neuroprotective effects of vanillin may be mediated through both anti-neuroinflammatory and anti-apoptotic activities.

### Vanillic acid

Vanillic acid (4-hydroxy-3-methoxybenzoic acid) is an oxidized form of vanillin and can be isolated from Tianma. Vanillic acid has antioxidant activity. Vanillic acid was found to reduce hydrogen peroxide-induced death and ROS in human SH-SY5Y cells (Gay et al., 2018). It can maintain the levels of FoxO3a, manganese superoxide dismutase (MnSOD), and catalase, contributing to its anti-oxidative stress activity. It also has the potential to be used in combination with clinical medications to provide better neuroprotective effects for PD. According to one study in a rotenone-induced PD rat model (Sharma et al., 2021), vanillin combined with levodopa-carbidopa co-treatment was found to significantly improve rotenone-induced motor deficits and reduce various oxidative stress indicators such as increased lipid peroxidation and decreased GSH and catalase in the brain. This combined treatment also increased dopamine level in rotenone-treated rats. However, vanillic acid treatment alone did not provide any effects. *In silico* prediction showed that vanillic acid could not cross the BBB (Salau et al., 2020), hinting that vanillic acid may have unknow mechanism to enhance the effects of levodopa-carbidopa.

### Vanillyl alcohol

Vanillyl alcohol (4-Hydroxy-3-methoxybenzyl alcohol) is a bioactive compound of Tianma that has antioxidant properties (Bezerra-Filho et al., 2019). It has been reported to have neuroprotection in a neurotoxin-induced PD cell model. Vanillyl alcohol exhibited anti-apoptotic effects in MPP^+^-induced dopaminergic MN9D cells by significantly reducing the ratio of Bax/Bcl-2 and the activation of PARP (Kim et al., 2011). It also reduced ROS level in MPP^+^-treated MN9D cells. The results of electron spin resonance spectrometry further supported that vanillyl alcohol is a strong antioxidant with scavenging activity for DPPH and alkyl radicals. It illustrates that the protective activity of vanillyl alcohol against MPP^+^-induced cell injury is dependent on its antioxidant ability; however, the activity of vanillyl alcohol lacks evidence in PD animal models.

### Anisalcohol

Anisalcohol (p-methoxybenzyl alcohol or 4-methoxybenzyl alcohol) is a phenolic compound that can be isolated from Tianma and is widely used in food flavoring and fragrances (Duan et al., 2013). One report indicated that anisalcohol was found to have anti-inflammatory effects in LPS-activated BV-2 microglia. LPS-stimulated microglia become M1 type and release pro-inflammatory factors such as TNF-α, IL-6, and PGE_2_, leading to neurotoxicity. Treatment with anisalcohol selectively modulates microglia polarization and also reduces LPS-induced pro-inflammatory factor production (Xiang et al., 2018). Notably, the generation of anti-inflammatory cytokine IL-10 and transforming growth factor -β (TGF-β) was enhanced by anisalcohol treatment in LPS-activated BV-2 cells (Xiang et al., 2018). They also performed flow cytometry using antibodies against CD16/32 and CD206 to confirm the polarization states of microglia and found that anisalcohol treatment could promote microglia to become M2 type microglia, which serve a protective role in the brain. In a mechanistic study, anisalcohol was found to display anti-inflammatory activity by inhibiting JNK/NF-κB activation, supporting the anti-neuroinflammation activity of anisalcohol.

## Discussion

Dopaminergic neuron death, α-Syn aggregation, and neuroinflammation are major characteristics of PD and can be used as key indicators for evaluating the efficacy of promising PD drugs. In this review, we assessed the effects of five constituents of Tianma on these major features of PD (Table 1). Although all these components are phenolic compounds with similar structures, their bioactivities are different in PD models. Gastrodin and vanillyl alcohol are reported to have protective effects against MPP^+^-induced cytotoxicity by upregulating Bcl-2 protein and thereby inhibiting the apoptotic pathway in PD cell models (Kumar et al., 2013; Jiang et al., 2014; Gay et al., 2018). Comparing their working concentrations (Table 2), gastrodin (1,5 and 25 μM) works at lower concentrations than vanillyl alcohol (10 and 20 μM). In the H_2_O_2_ cell model, vanillic acid treatment maintains the levels of SOD2, catalase, and Bcl-2 protein expression (Kim et al., 2011), suggesting that vanillic acid works through mediating oxidative stress-induced cell death. However, evidence to support the protective effect of vanillic acid against MPP^+^-induced cytotoxicity is still lacking. In PD animal models, only gastrodin and vanillin exert neuroprotective effects, probably due to their ability to cross BBB. According to mechanistic studies, gastrodin can enhance Nrf2-mediated intracellular antioxidant system, which is critical in maintaining mitochondrial functions under oxidative damage (Jiang T et al., 2020). Therefore, current evidences do support that gastrodin is able to reduce PD neurotoxin-induced mitochondrial dysfunction and oxidative stress as well as enhance the survival of neurons in different PD models. In contrast, there is no report regarding the action mechanism of vanillin and further study is needed to identify what contributes to the neuoprotective effects of vanillin.

**TABLE 1:** The effects of bioactive Tianma compounds on various *in intro* and *in vivo* PD models.

Active compounds	Model	Inducer	Effects	References
Gastrodin 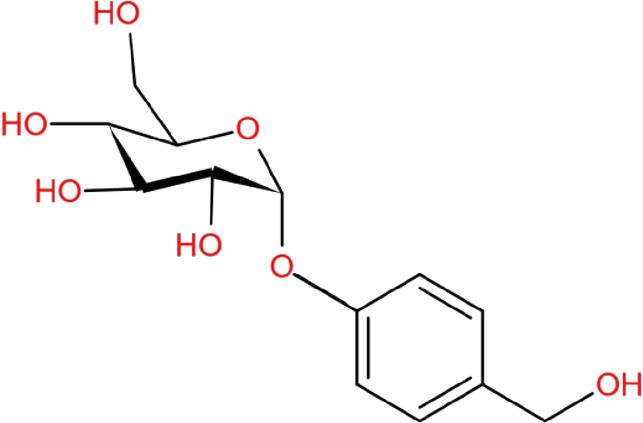	*In vitro*	MPP^+^	Cell viability↑, DAPPH↓, Alkyl↓, ROS↓, SOD↑, Bcl-2↑, Bax↓, Caspase 3↓, PARP cleavage↓	Kumar et al. (2013)
	SH-SY5Y cells			
	*In vivo*	MPTP	TH↑, GFAP↑, Motor deficits↓	Kumar et al. (2013)
	Male 57BL/6 mice			
	*In vitro*	Rotenone	p-Cx43↓	Wang et al. (2014)
	Primary astrocyte			
	*In vivo*	Rotenone	p-Cx43↓	Wang et al. (2014)
	Lewis rat			
	*In vitro*	MPP^+^	Cell viability↑, Bcl-2↑, Bax↓, ROS↓, Nfr2 nuclear translocation↑, HO-1↑	Jiang et al. (2014)
	SH-SY5Y cells			
	*In vivo*	MPTP	HO-1↑, SOD↑, GSH↑, p-ERK↓, Nrf2 nuclear translocation↑	Wang et al. (2014)
	Male C57BL/6 mice			
	*In vivo*	6-OHDA	Motor balance↑, MPO↓, NO↓, TAC↑	Haddadi et al. (2018)
	Male Wistar rats			
	*In vivo*	6-OHDA	chemotaxis↑, DAF-2↑	Yan et al. (2019)
	C. elegans			
	*In vitro*	H_2_O_2_	GSH↑, GPX4↑, ROS↓	Jiang T et al. (2020)
	Rat Glioma Cell Line C6			
	*In vivo*	PINK1 gene mutant	Lifespan↑, locomotor ability↑, antioxidant activity↑	He et al. (2021)
	Drosophila			
	*In vivo*	Rotenone	TNF-α↓, IL-1β↓	Li et al. (2012)
	Male Wistar rats			
	*In vitro*	LPS	iNOS↓, TNF-α↓, cyclin-D1↓, Wnt/β-catenin signaling↓	Yao et al. (2019)
	BV-2 microglia			
	*In vitro*	LPS	iNOS↓, COX2↓, TNF-α↓, IL-1β↓, NF-κB↓, p-MAPKs↓	Dai et al. (2011)
	BV-2 microglia			
Vanillin 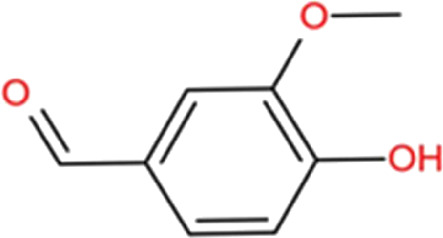	*In vivo*	6-OHDA	Tight contralateral rotations upon apomorphine challenge↓, dopamine concentrations↑	Abuthawabeh et al. (2020)
	Male Wistar Rats			
	*In vitro* microglia	LPS	p-MAPKs↓, p-NF-Κb↓, iNOS↓, COX-2↓, IL-1β↓, TNF-α↓, IL-6↓	Kim et al. (2019)
	*In vivo*	Rotenone	Motor symptoms↓, Non-Motor symptoms↓, Bax↓, caspase-3↓, Caspase-8↓, Caspase-9↓, Bcl-2 ↑, Cyt-C↑	Dhanalakshmi et al. (2016)
	Male Albino Wistar rats			
	*In vivo*	LPS	iNOS↓, COX-2↓, IL-1β↓, IL-6↓	Yan et al. (2017)
	Male Wistar rats			
Vanillic acid 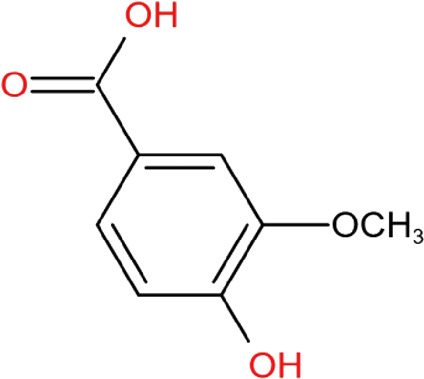	*In vivo*	Rotenone	GSH↑, SAG↑, motor deficit↓, TBARS↓, CAT↑	Sharma et al. (2021)
	Sprague Dawley rats			
	*In vitro*	H_2_O_2_	ROS↓, SIRT1↑, FoxO↑, Bcl-2↑	Gay et al. (2018)
	SH-SY5Y			
Vanillyl Alcohol 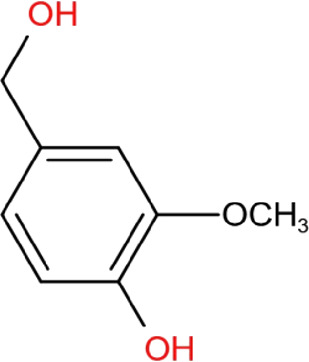	*In vitro*	MPP^+^	ROS↓, Bax/Bcl↓, PPAR cleavage↓	Kim et al. (2011)
	MN9D cells			
Anisalcohol 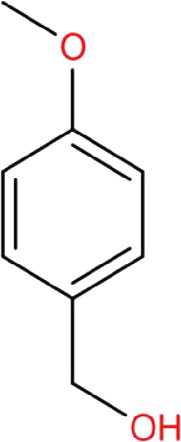	*In vitro*	LPS	Cell viability↑, NO↓, TNF-α↓, PCE_2_↓, TGF-β↑, p-JNK↓, CD16/32↓, CD206↑, p-NF-κB p65↓	Xiang et al. (2018)

**TABLE 2:** The working concentration of anti-apoptosis of Tianma compounds in cell models.

Compound name	Cell model	Working concentration	References
Gastrodin	MPP^+^/SH-SY5Y Cells	1,5,25 μM	Kumar et al. (2013) Jiang et al. (2014)
Vanillic acid	H_2_O_2_/SH-SY5Y Cells	1.5 μM	Gay et al. (2018)
Vanillyl Alcohol	MPP^+^/MN9D Cells	10,20 μM	Kim et al. (2011)

Gastrodin, vanillin, and anisalcohol are reported to reduce microglial activation and pro-inflammatory cytokine generation (Table 3). In the LPS/BV-2 cells, the working concentrations of anisalcohol (0.1, 1, 10 μM) were found to be much lower than those of vanillin (100, 200, 300, 400 μM) and gastrodin (20, 30, 40, 60 μM). Vanillin and gastrodin mediate anti-inflammatory activity *via* inhibiting LPS-induced phosphorylation of MAPK and NF-κB. Anisalcohol also inhibits p65 phosphorylation of NF-κB and JNK phosphorylation, while anisalcohol can significantly inhibit the M1 microglia marker CD16/32 and promote M2 microglia CD206 expression. These evidences support that anisalcohol has anti-inflammatory effects at the cellular level. However, there is no report regarding anti-neuroinflammation of anisalcohol in animal models. Notably, anisalcohol can promote microglia to become M2 type microglia. It is possible that gastrodin and vanillin also have similar effects on the changes of microglia polarisations. Both gastrodin and vanillin display *in vivo* anti-neuroinflammation activity, but the working concentration of gastrodin (200 mg/kg) is far higher than vanillin (20 mg/kg). The possible reason is that *in vivo* rapid metabolism of gastrodin into 4-hydroxybenzyl alcohol (4-HBA) may reduce its application *in vivo* because 4-HBA exhibits limited biological activity (Liu et al., 2018). Therefore, maintaining the *in vivo* stability of gastrodin may be a critical issue to solve for future applications.

**TABLE 3:** The working concentration of anti-inflammation of Tianma compounds.

Compound name	Cell model	Working concentration	References
Gastrodin	BV-2 microglia	30,40,60 μM	Dai et al. (2011)
Gastrodin	BV-2 microglia	20,40 μM	Yao et al. (2019)
Vanillin	BV-2 microglia	100,200,300,400 μM	Kim et al. (2019)
Anisalcohol	BV-2 microglia	0.1,1,10 μM	Xiang et al. (2018)

Gastrodin is the only candidate that may clear the accumulation of α-Syn. Strikingly, gastrodin is reported to exhibit anti-aging activity in Drosophila (He et al., 2021). Since aging is the major driver of PD development, it would be interesting examine whether this activity can be observed in other species. Compared with other compounds, gastrodin not only decreases three major PD features but also slows aging, providing vital support for its potential for PD.

These studies still have limitations because none of the current experimental models can mimic all clinical features of PD (Ke et al., 2021). The classical neurotoxin (MPTP, 6-OHDA, and rotenone)-induced PD animal models can rapidly generate the loss of dopaminergic neurons and neuroinflammation without disease progression. These *in vivo* models help define the benefits of drug candidates to reduce PD symptoms. Cell models are treated with neurotoxins to induce oxidative stress, mitochondria dysfunction, and cell death. LPS-induced BV-2 microglia activation is commonly used for investigating neuroinflammation. Undoubtedly, these *in vitro* models can mimic some PD features for drug screening and mechanistic study. However, a poor understanding of PD etiology and pathogenesis still limits us from identifying the suitability of drug candidates. In addition, these models fail to reflect the impact of aging. Thus, it may be necessary to evaluate the effects of drug candidates *via* using more diverse models such as transgenic animal models and neurons derived from induced pluripotent stem cells of human PD patients; alternatively, establishing more precise models to mimic PD development may help assess the effects of potential drug candidates.

There are many similarities between Tianma and Ganoderma. Both have complicated components, but have been used for centuries in Chinese medicine, which may support their safety as therapeutics. In *vitro* and *in vivo* studies, they can improve motor symptoms and various major PD features (Zhang et al., 2011; Ren et al., 2019). In toxicology experiments, it was found that a 28-day oral intake of Tianma water extract (8,065 mg/kg body weight) did not have adverse effects on mortality, body weight, organ weight, behavior, hematology, and clinical biochemistry (Lu et al., 2020). Notably, gastrodin was found in a clinical study (NCT00297245) to prevent cognitive decline after cardiac surgery with cardiopulmonary bypass. Results indicated that gastrodin is an effective and safe drug for preventing the neurocognitive decline in patients (Zhang et al., 2011). These studies support that Tianma and its active compounds are safe and have the potential to relieve neurological symptoms.

## Conclusion

So far, the exact pathogenic mechanisms of PD are not well understood, thereby resulting a lack of effective drugs for preventing PD development. The bioactive components of Tianma have various positive effects on dopaminergic neuron death, α-synuclein aggregation, and neuroinflammation, further supporting the therapeutic potential of Tianma for PD. Of these, gastrodin shows greatest potential as it reduces the characteristics of PD and displays anti-aging activity in preclinical studies. Further studies on Tianma and its major compounds are warranted to fully understand the toxicological profiles, pharmacokinetics, and the underlying molecular mechanisms of these naturally occurring compounds and their potential for the prevention and treatment of PD.
